# Curcumin Pretreatment Alleviates Early Mannheimia Haemolytica-Induced Pulmonary Injury in Mice and Is Associated with Changes in Gut Microbial Composition

**DOI:** 10.3390/microorganisms14081764

**Published:** 2026-08-11

**Authors:** Fangfang Zhao, Yixin Huang, Shifeng Wu, Yong Fu, Xueyong Zhang, Zhi Li, Xin An, Kun Zhang, Liuhong Shen, Suizhong Cao, Huanrong Zhang, Xiuying Shen, Shumin Yu

**Affiliations:** 1 College of Veterinary Medicine, Sichuan Agricultural University, Chengdu 610041, China; 2 Qinghai Academy of Animal Science and Veterinary, Qinghai University, Xining 810016, China; 3 College of Animal and Veterinary Sciences, Southwest Minzu University, Chengdu 610041, China

**Keywords:** curcumin, Mannheimia haemolytica, early acute pulmonary injury, preventive pretreatment, NF-κB/NLRP3-related proteins, gut microbiota

## Abstract

Mannheimia haemolytica (Mh) is a major bovine respiratory pathogen that can trigger marked inflammatory and barrier injuries. This study evaluated the protective effect of oral curcumin pretreatment in a proof-of-concept mouse model of early Mh-induced acute pulmonary injury. Mice received curcumin at 50 or 150 mg/kg/day for seven days before an intranasal Mh challenge and were evaluated 12 h after infection. Curcumin pretreatment was associated with lower lung wet-to-dry ratios, bronchoalveolar lavage fluid protein concentrations, histological injury scores, and serum pro-inflammatory cytokine levels. It was also associated with reduced abundance or phosphorylation of NF-κB/NLRP3-related proteins, increased expression of Nrf2-associated antioxidant proteins, reduced apoptosis, and preservation of the tight-junction protein ZO-1. In addition, curcumin pretreatment was associated with changes in fecal microbial composition and intestinal short-chain fatty acid profiles, including partial recovery of propionic acid. Because this study did not include a curcumin-only group, post-infection treatment, bacterial-burden measurements, pathway-perturbation experiments, or microbiota-causality experiments, the findings support prophylactic host-protective associations in an early mouse model rather than antibacterial activity, therapeutic efficacy after established infection, or causal gut–lung mechanisms. Validation in bovine models is required before veterinary application.

## 1. Introduction

Mannheimia haemolytica (Mh) is a major contributor to ruminant animals respiratory disease complex (BRDC) and can cause severe neutrophilic inflammation, edema, hemorrhage, and epithelial injury in cattle [1,2]. Although antimicrobial therapy remains central to disease control, antibiotic resistance and the persistence of damaging host inflammatory responses support investigation of complementary host-directed strategies [3,4]. Mh-associated pulmonary injury involves dysregulated inflammation, oxidative stress, apoptosis, and disruption of epithelial barrier integrity, with NF-κB/NLRP3- and Nrf2-related signaling proteins implicated in these processes [5,6,7]. The gut–lung axis may also influence respiratory inflammation, although compositional associations alone do not establish causality [8,9]. In the present work, a C57BL/6J mouse model was used as a controlled proof-of-concept model of early acute pulmonary injury and host response; it was not intended to reproduce all features of bovine Mannheimia pneumonia.

**Figure 1:**
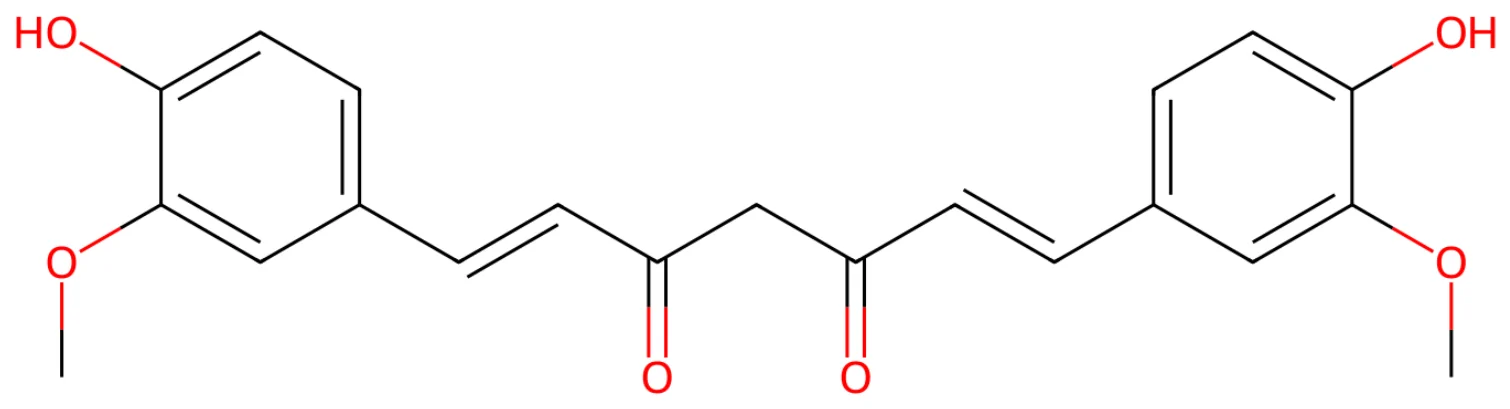
Chemical structure of curcumin.

Curcumin (Cur), a natural polyphenol derived from Curcuma longa, has anti-inflammatory, antioxidant, anti-apoptotic, and barrier-associated activities in several experimental systems [10,11,12,13,14,15,16]. Its broad biological profile makes it a candidate for host-directed prevention of excessive tissue injury. However, curcumin has poor aqueous solubility and limited oral bioavailability, and its effects can depend strongly on formulation and exposure [17,18]. The molecular structure of curcumin is shown in Figure 1.

This study evaluated whether seven days of oral curcumin pretreatment would attenuate early pulmonary injury measured 12 h after an Mh challenge. We assessed lung edema and histopathology, systemic inflammatory markers, pathway-associated proteins, apoptosis, epithelial junction proteins, fecal bacterial composition, and intestinal short-chain fatty acids. The design was prophylactic rather than post-infection therapeutic, and the molecular and microbiota measurements were interpreted as associations rather than direct evidence of pathway or gut–lung causality.

## 2. Materials and Methods

### 2.1. Animal Grouping and Treatment

C57BL/6J mice (6–7 weeks old, 18–20 g) were obtained from Sichuan Dashuo Animal Co., Ltd. (Chengdu, China) and housed under specific pathogen-free conditions (25 ± 1 °C; relative humidity, 40–60%; 12 h light/dark cycle) with ad libitum access to autoclaved food and water. Thirty-two mice were randomly assigned to four groups (n = 8/group): control (Con), Mh, low-dose curcumin plus Mh (Cur-D), and high-dose curcumin plus Mh (Cur-G). Curcumin powder (HY-N0005, MCE) was suspended in sterile saline and mixed thoroughly immediately before gavage. Con and Mh mice received sterile saline, whereas Cur-D and Cur-G mice received 2.5 or 7.5 mg/mL of curcumin at 0.2 mL/10 g body weight once daily for seven consecutive days, corresponding to 50 and 150 mg/kg/day, respectively. This schedule represents preventive pretreatment. Mh, Cur-D, and Cur-G mice were then challenged intranasally with 50 μL of M. haemolytica serotype A1 (4 × 10^8^ CFU/mL in sterile PBS). Mice were fasted for 6 h before euthanasia and were euthanized 12 h after challenge by intraperitoneal sodium pentobarbital (30 mg/kg); death was confirmed by absence of corneal and pedal-withdrawal reflexes. The 12 h endpoint was selected to capture an early acute host response and does not represent established or resolving pneumonia. All procedures were approved by the Institutional Animal Care and Use Committee of Sichuan Agricultural University (SICAU2024-019) and followed ARRIVE guidelines.

### 2.2. The Determination of the W/D Ratio

Following euthanasia, the thoracic region was disinfected with 70% ethanol. The thoracic cavity was aseptically opened to excise the left lung, which was immediately rinsed with sterile phosphate-buffered saline (PBS) to remove surface contaminants. Residual moisture was absorbed using sterile filter paper, and the tissue was transferred to a pre-labeled 1.5 mL sterile microcentrifuge tube for gravimetric analysis. The wet weight of the lung was recorded using an analytical balance (resolution: 0.1 mg).

For dehydration, samples were placed in a forced-air drying oven maintained at 80 °C for 48 h until constant mass was achieved (defined as <0.2% weight variation between successive measurements). The dry weight was subsequently documented. Pulmonary edema severity was quantified through the wet-to-dry weight ratio (W/D ratio), calculated as: W/D ratio = Wet weight (g)/Dry weight (g).

### 2.3. The Determination of BALF Concentration

Following euthanasia, tracheal isolation was performed under aseptic conditions. A sterile 24-gauge indwelling catheter was inserted into the tracheal lumen, and bronchoalveolar lavage (BAL) was conducted using three sequential 500 μL aliquots of ice-cold sterile phosphate-buffered saline (PBS, pH 7.4). The recovered BAL fluid (BALF) was pooled and immediately centrifuged at 3000× *g* for 10 min in a pre-cooled rotor (4 °C).

The supernatant was aliquoted into RNase/DNase-free microcentrifuge tubes and stored at −80 °C pending analysis. Total protein concentration in BALF was quantified using a bicinchoninic acid (BCA) assay kit (Pierce™, Thermo Fisher Scientific, Waltham, MA, USA) according to the manufacturer’s protocol, with absorbance measured at 562 nm on a microplate reader (BioTek Synergy H1, Winooski, VT, USA). Protein levels were normalized to BALF recovery volume and expressed as μg/mL, serving as a biomarker of alveolar-capillary permeability.

### 2.4. Histopathology

Lung specimens were harvested 12 h after Mh challenge during the early acute-injury phase. The right superior pulmonary lobe was immersion-fixed in 10% neutral buffered formalin (pH 7.4) for 24 h at 4 °C, followed by sequential ethanol dehydration (70%, 80%, 95%, and 100% *v*/*v*) and paraffin embedding using a tissue processor (Leica ASP300, Nussloch, Germany). Serial 4-μm sections were mounted on poly-L-lysine-coated slides (Thermo Scientific) and stained with hematoxylin (Harris modified formula) and eosin Y (H&E) through an automated stainer (Sakura Tissue-Tek Prisma, Sakura Finetek Japan Co., Ltd., Tokyo, Japan). Histopathological evaluation was conducted by two board-certified pathologists blinded to experimental groups using brightfield microscopy (Nikon Eclipse Ni-E, Tokyo, Japan) with DS-Ri2 camera documentation.

A semi-quantitative lung injury scoring system was implemented based on four histopathological parameters: alveolar capillary congestion, intra-alveolar hemorrhage, neutrophilic infiltration (perivascular/peribronchial), and septal thickening/hyaline membrane formation. Each parameter was graded on a 0–4 severity scale: 0 = no involvement/normal architecture, 1 = <25% involvement, 2 = 25–50% involvement, 3 = 50–75% involvement, and 4 = >75% involvement. The composite injury score was calculated as the sum of individual parameter scores (maximum 16 points per specimen), with higher cumulative scores indicating greater histological damage. All measurements were normalized to 10 randomly selected high-power fields per section.

### 2.5. The Measurement of Immunoglobulins and Cytokines

Blood was allowed to clot for 1 h at room temperature and centrifuged at 1000× *g* for 10 min to obtain serum. Serum IL-1β, IL-4, IL-8, TNF-α, D-lactate, IgA, IgG, and IgM were measured using commercially available kits (Jiangsu MEIMIAN Industrial Co., Ltd., Yancheng, China) according to the manufacturer instructions. D-lactate was included as a systemic indicator associated with microbial metabolism and intestinal barrier disturbance; it was not interpreted as a direct measure of pulmonary inflammation or lung barrier integrity.

### 2.6. Western Blotting Assay

Lung tissue specimens (≈100 mg) were homogenized in ice-cold RIPA lysis buffer (Thermo Scientific, 89901) supplemented with Protease/Phosphatase Inhibitor Cocktail (Thermo Scientific, 78446) at a 1:10 (*w*/*v*) ratio using a Polytron PT 1200E homogenizer (Kinematica, Malters, Switzerland). The homogenate underwent three cycles of ultrasonication (10-s pulse/30-s rest on ice; 40% amplitude; Sonics VCX750, Newtown, CT, USA) to ensure complete cellular disruption. Lysates were clarified by centrifugation at 10,000× *g* for 15 min at 4 °C (Eppendorf 5430R, Hamburg, Germany), and supernatants were quantified using a BCA Protein Assay Kit (Pierce, 23227, Waltham, MA, USA) with bovine serum albumin standards.

Protein extracts (30 μg/lane) were mixed with 4× Laemmli buffer containing 5% β-mercaptoethanol, heated at 95 °C for 5 min, separated on 10% Tris-glycine SDS-PAGE gels, and transferred to 0.45 μm PVDF membranes. Membranes were blocked in 5% non-fat milk in TBST and incubated overnight at 4 °C with antibodies against ASC, cleaved caspase-1, GSDMD-N, NLRP3, TLR4, phosphorylated NF-κB p65, total NF-κB p65, cleaved caspase-3, Bax, Bcl-2, NQO1, HO-1, Keap1, phosphorylated Nrf2, total Nrf2, occludin, claudin-1, ZO-1, and β-actin.

After washing, membranes were incubated with HRP-conjugated secondary antibodies (1:5000) for 1 h at 25 °C. Bands were visualized using SuperSignal West Pico PLUS (Thermo Fisher Scientific, Waltham, MA, USA) substrate and quantified with Image Lab 6.1. Non-phosphorylated target proteins were normalized to β-actin; phosphorylated NF-κB p65 and phosphorylated Nrf2 were normalized to their corresponding total proteins. For in vivo immunoblot quantification, each point represents one independent animal-derived biological sample (n = 3/group). For the BT-cell experiments, each point represents one independently conducted cell-culture experiment (three independent experiments).

### 2.7. Flow Cytometry

Lung tissues were harvested, mechanically homogenized in ice-cold phosphate-buffered saline (PBS), and treated with Trypsin for 5 min at 4 °C. After two washes with PBS, the cell pellets were resuspended in fresh PBS. Pneumonocyte apoptosis was quantified using an Annexin V-FITC/PI Apoptosis Detection Kit (BD Biosciences, San Jose, CA, USA). Briefly, cell suspensions were incubated with 5 μL Annexin V-FITC and 5 μL propidium iodide (PI) in the dark for 15 min at room temperature (25 °C). Cells staining Annexin V^+^/PI^−^ and Annexin V^+^/PI^+^ were classified as early apoptotic and late apoptotic populations, respectively. Flow cytometry (FCM) was performed on a BD FACSCanto II system (BD Biosciences), and data were analyzed using FlowJo software (v10.6.2, TreeStar, Ashland, OR, USA).

### 2.8. Fecal Collection and 16S rRNA Sequencing Analysis

Fecal samples were collected from individual mice during the three days before sacrifice (n = 8/group), immediately frozen in liquid nitrogen, and stored at −80 °C. Microbial DNA was extracted using the E.Z.N.A. Soil DNA Kit (Omega Bio-tek, Inc., Norcross, GA, USA) with bead beating. The V3-V4 region of the bacterial 16S rRNA gene was amplified using primers 338F and 806R, purified, normalized, and sequenced as 2 × 300-bp paired-end reads on an Illumina MiSeq platform with a 15% PhiX spike-in. Each biological sample represented one mouse. PICRUSt2-derived KEGG profiles were treated as predictions of functional potential based on 16S composition and not as direct measurements of microbial gene expression or metabolic activity.

### 2.9. Quantification of Intestinal Short-Chain Fatty Acids

Intestinal samples were processed for volatile fatty acid (VFA) profiling through a validated derivatization-GC/MS protocol. Briefly, tissue homogenates (100 mg/mL in PBS) were centrifuged at 300× *g* for 10 min at 4 °C (Eppendorf 5430R). Supernatants underwent protein precipitation by adding 25% (*w*/*v*) metaphosphoric acid (Sigma-Aldrich, 79610, St. Louis, MO, USA) at a 9:1 (*v*/*v*) ratio, followed by vortex mixing (30 s) and incubation at 4 °C for 30 min. After secondary centrifugation (12,000× *g*, 15 min, 4 °C), clarified extracts were filtered through 0.22 μm PTFE membranes (Millipore, SLGV033RB, Billerica, MA, USA) into pre-labeled GC vials with low-volume inserts (Agilent, 5183-2087, Santa Clara, CA, USA).

Analyses were performed on a Shimadzu GCMS-QP2020 NX system equipped with a DB-FFAP capillary column (30 m × 0.25 mm × 0.25 μm; Agilent J&W 122-3232, Santa Clara, CA, USA). The temperature program included: Initial: 60 °C (3 min hold), Ramp 1: 20 °C/min to 220 °C (1 min hold), and Total run time: 12 min. Helium carrier gas flow was maintained at 1.2 mL/min with splitless injection (1 μL). Mass spectrometry operated in electron ionization mode (70 eV), scanning *m/z* 40–450. Data acquisition utilized LabSolutions GCMS software (v5.99, Shimadzu, Kyoto, Japan), with compound identification based on: Retention index alignment (±5 s) against C4-C18 n-alkane standards, Spectral similarity (≥85%) to NIST 20 library, and Co-elution with authentic reference standards (Sigma-Aldrich). Quantitation employed 2-ethylbutyric acid (50 μg/mL) as an internal standard (IS). Analyte concentrations were calculated via linear calibration curves (R^2^ > 0.995) using peak area ratios (analyte/IS), normalized to tissue wet weight.

### 2.10. Cell Culture and Treatment

Bovine turbinate (BT) cells were cultivated in Dulbecco’s Modified Eagle’s Medium (DMEM; Gibco, Grand Island, NY, USA) under controlled conditions of 37 °C and 5% CO_2_. The culture medium was fortified with 10% fetal bovine serum (FBS; HyClone, Logan, UT, USA) and a 1% solution of 100 μg/mL penicillin-streptomycin antibiotic mixture (HyClone, Logan, UT, USA). Mh was dissolved in a serum-free medium for subsequent treatment of BT cells. Experimental groups were designed as follows: Control (Con), Mh-treated group, Mh + Cur (40 μM) group, and Mh + Cur (80 μM) group. Cur was introduced into the culture medium 2 h, followed by the continuous treatment of Mh for 24 h.

### 2.11. Statistical Analysis

Data are presented as mean ± SD. Each mouse, animal-derived tissue sample, fecal sample, or independently seeded cell experiment was treated as a biological replicate. Technical measurements, when present, were averaged before statistical analysis. Sample sizes are stated in the figure legends: n = 6 animals/group for lung injury indices, serum analytes, and intestinal short-chain fatty acids; n = 3 independent biological samples/group for in vivo immunoblotting and flow cytometry; n = 8 fecal samples/group for 16S rRNA sequencing; and three independent experiments for in vitro assays. Normality and homogeneity of variance were assessed using Shapiro–Wilk and Brown–Forsythe tests. Group differences were analyzed by one-way ANOVA followed by Tukey multiple-comparison tests. Analyses were performed in GraphPad Prism 9.4.1. Statistical significance was defined as *p* < 0.05. The original unsupported statement regarding a priori statistical power was removed.

## 3. Results

### 3.1. Curcumin Pretreatment Attenuates Early Pulmonary Injury After Mh Challenge

**Figure 2:**
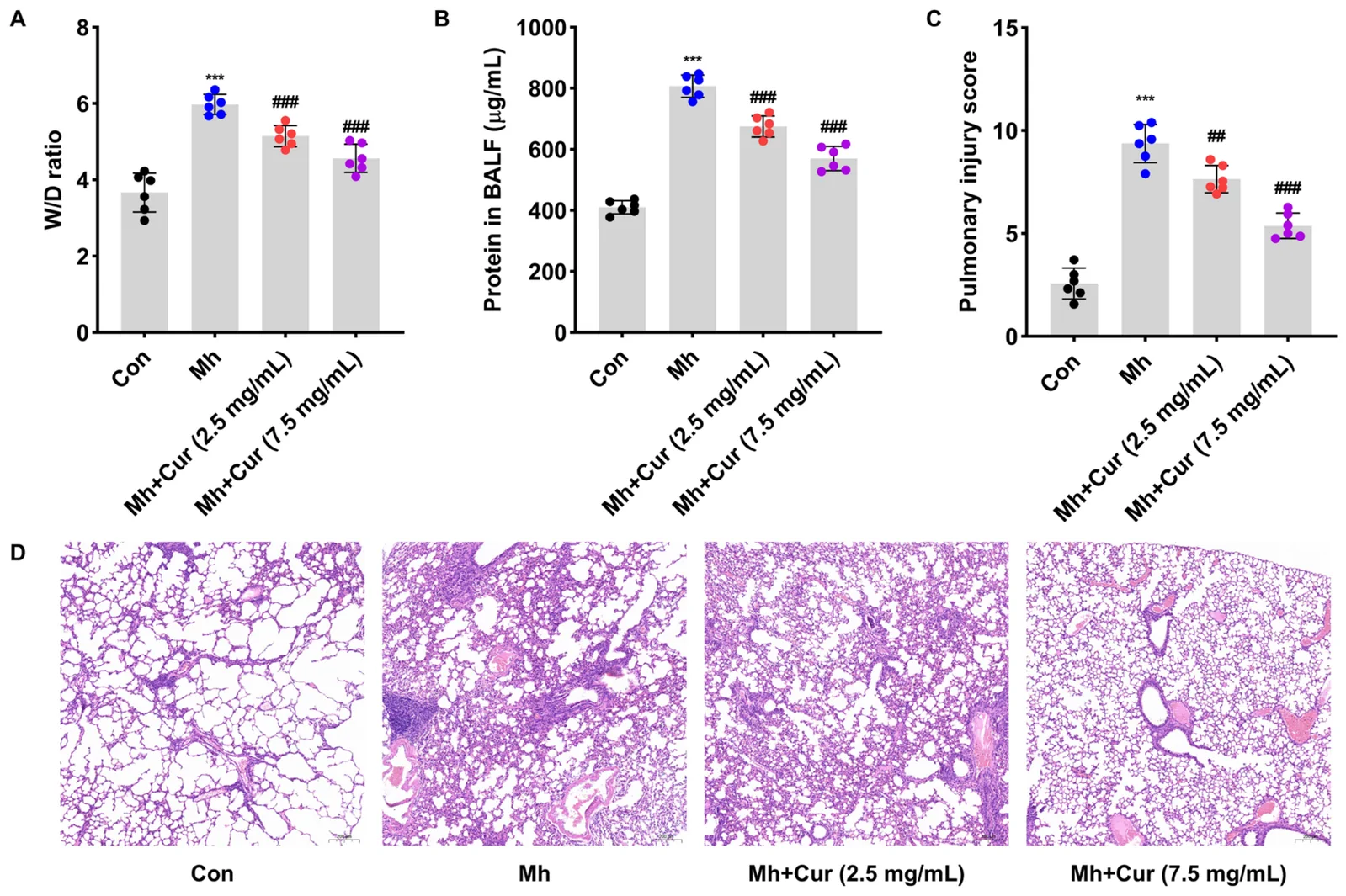
Curcumin pretreatment attenuates early pulmonary injury after Mh challenge. (**A**) Lung wet-to-dry weight ratio. (**B**) BALF protein concentration. (**C**) Representative H&E-stained lung sections (scale bar, 200 μm). (**D**) Lung-injury score. n = 6 independent animals/group. Data are means ± SDs. *** *p* < 0.001 versus Con; ## *p* < 0.01 and ### *p* < 0.001 versus Mh.

Mh challenge increased the lung wet-to-dry ratio, BALF protein concentration, and histological lung-injury score compared with the control group (Figure 2A–D). Curcumin pretreatment was associated with dose-dependent reductions in these early injury indices. Histological examination showed marked inflammatory-cell infiltration, hemorrhage, and congestion after Mh challenge, whereas these changes were less pronounced in the curcumin-pretreated groups. Because pulmonary bacterial burden was not measured, these findings indicate attenuation of host tissue injury but do not establish an antibacterial effect.

### 3.2. Curcumin Pretreatment Is Associated with Changes in Serum Immunoglobulins, D-Lactate, and Cytokines

**Figure 3:**
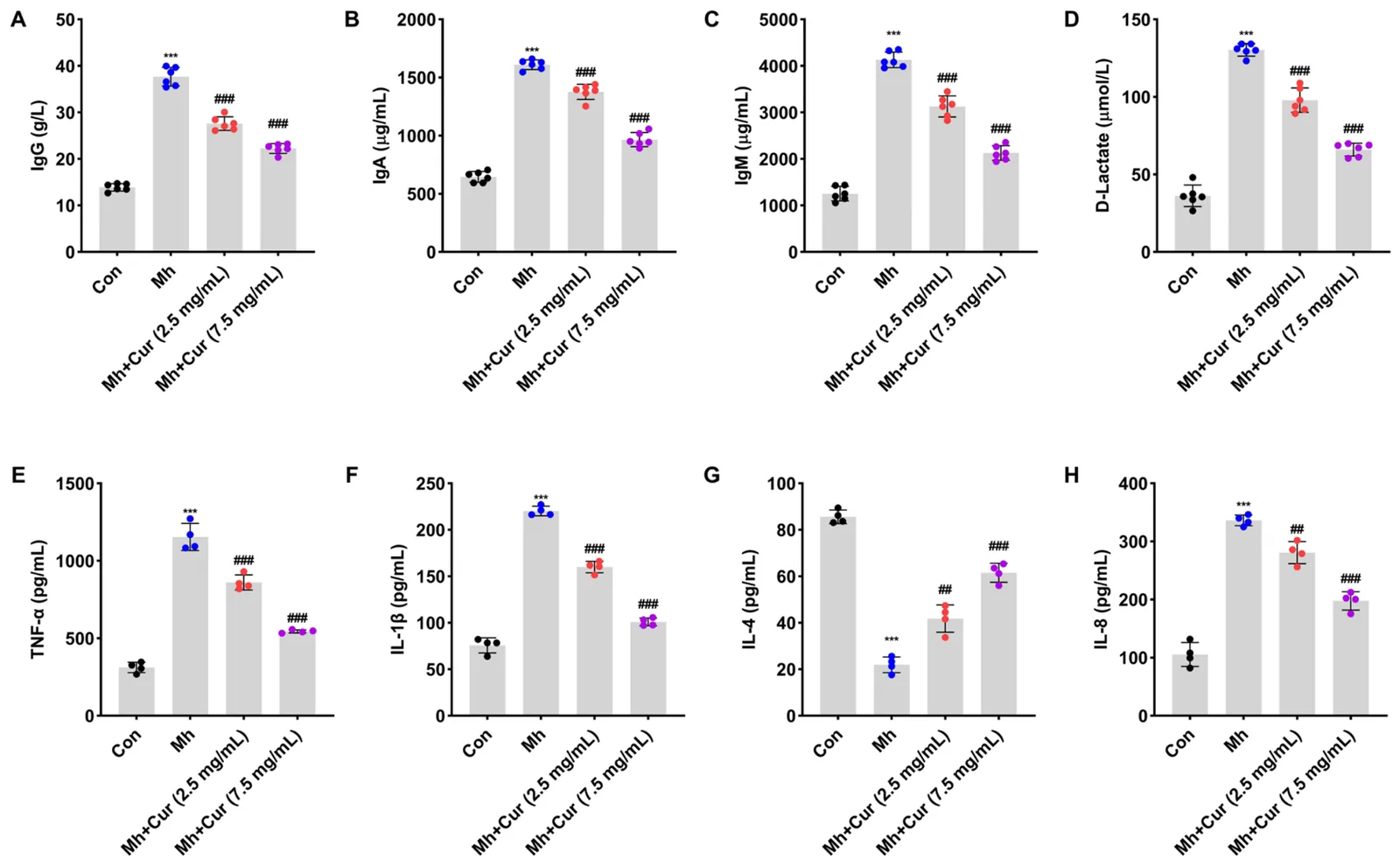
Curcumin pretreatment is associated with changes in serum immunoglobulins, D-lactate, and cytokines. Serum IgG (**A**), IgA (**B**), IgM (**C**), D-lactate (**D**), TNF-α (**E**), IL-1β (**F**), IL-4 (**G**), and IL-8 (**H**) were measured. n = 6 independent animals/group. Data are means ± SDs. *** *p* < 0.001 versus Con; ## *p* < 0.01 and ### *p* < 0.001 versus Mh.

Mh challenge increased serum IgG, IgA, IgM, and D-lactate concentrations, whereas curcumin pretreatment was associated with lower values (Figure 3A–D). These results indicate changes in systemic immune and gut-associated markers during the early response. D-lactate was interpreted cautiously as an indirect systemic indicator associated with intestinal barrier or microbial metabolic disturbance, not as proof of a causal gut–lung mechanism.

Serum TNF-α, IL-1β, and IL-8 increased and IL-4 decreased after the Mh challenge. Curcumin pretreatment was associated with partial normalization of these serum cytokines (Figure 3E–H). Because cytokines were measured in serum rather than BALF or lung homogenates, these findings primarily reflect systemic inflammatory changes and should not be interpreted as direct quantification of local pulmonary cytokine production.

### 3.3. Curcumin Pretreatment Is Associated with Reduced NF-κB/NLRP3-Related Inflammatory Protein Changes

**Figure 4:**
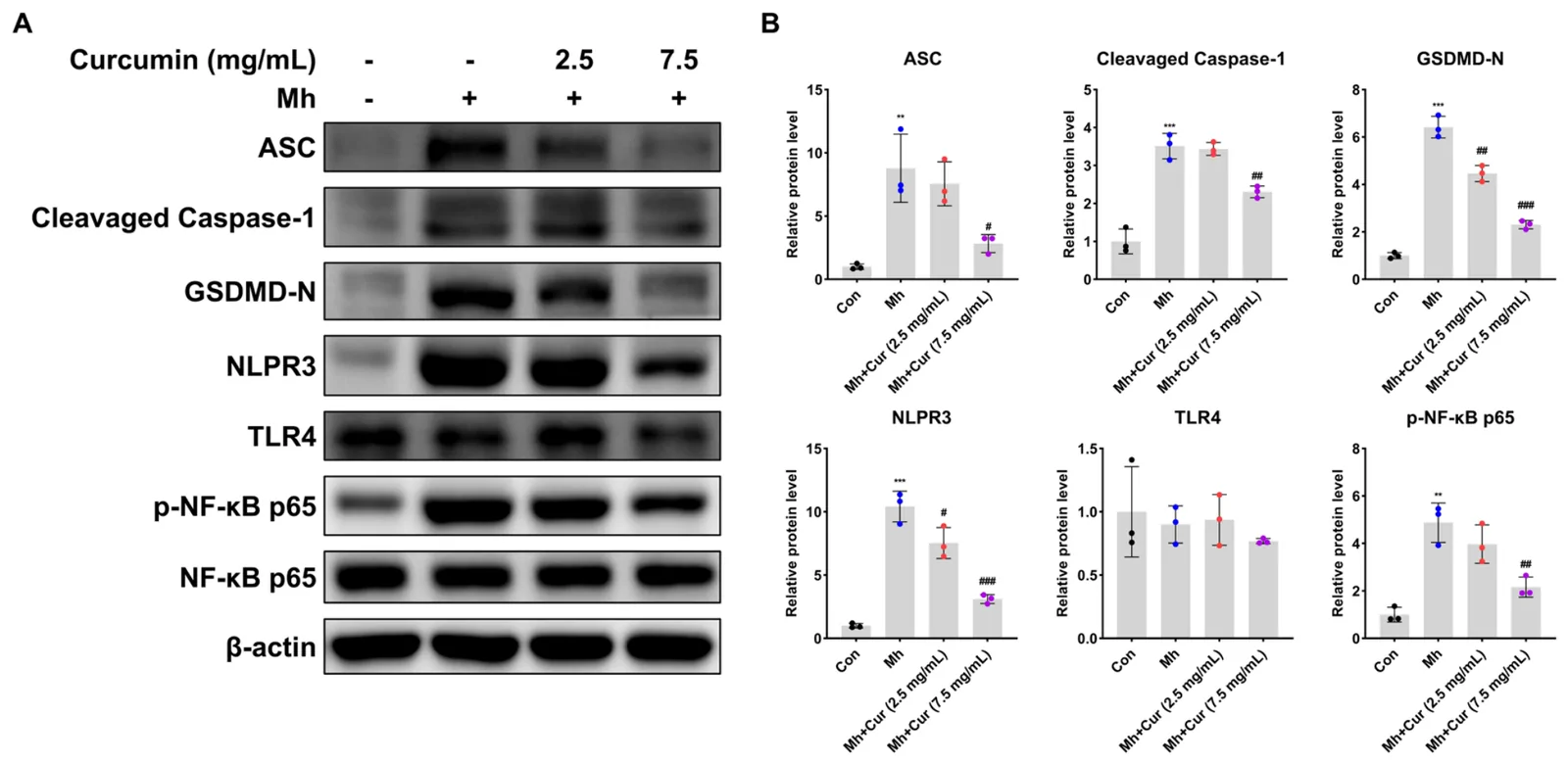
Curcumin pretreatment is associated with reduced inflammation-related protein changes in lung tissue. (**A**) Immunoblots for ASC, cleaved caspase-1, GSDMD-N, NLRP3, TLR4, phosphorylated NF-κB p65, total NF-κB p65, and β-actin. (**B**) Densitometric analysis. Non-phosphorylated targets were normalized to β-actin; phosphorylated NF-κB p65 was normalized to total NF-κB p65. n = 3 independent animal-derived biological samples/group. Data are means ± SDs. ** *p* < 0.01 and *** *p* < 0.001 versus Con; # *p* < 0.05, ## *p* < 0.01, and ### *p* < 0.001 versus Mh.

The Mh challenge was associated with increased abundance of ASC, cleaved caspase-1, GSDMD-N, NLRP3, and phosphorylated NF-κB p65 in lung tissue. Curcumin pretreatment was associated with lower levels of these inflammation-related proteins (Figure 4A,B). TLR4 abundance changed less consistently.

NF-κB activation can contribute to NLRP3 inflammasome priming and caspase-1-dependent inflammatory responses [19,20,21,22]. In the present study, the lower phosphorylated NF-κB p65 and inflammasome-related protein levels observed after curcumin pretreatment are consistent with reduced pathway activation. However, no pathway inhibitors, knockdown, overexpression, or rescue experiments were performed; therefore, the results do not establish that curcumin directly inhibited the NF-κB/NLRP3 axis.

**Figure 5:**
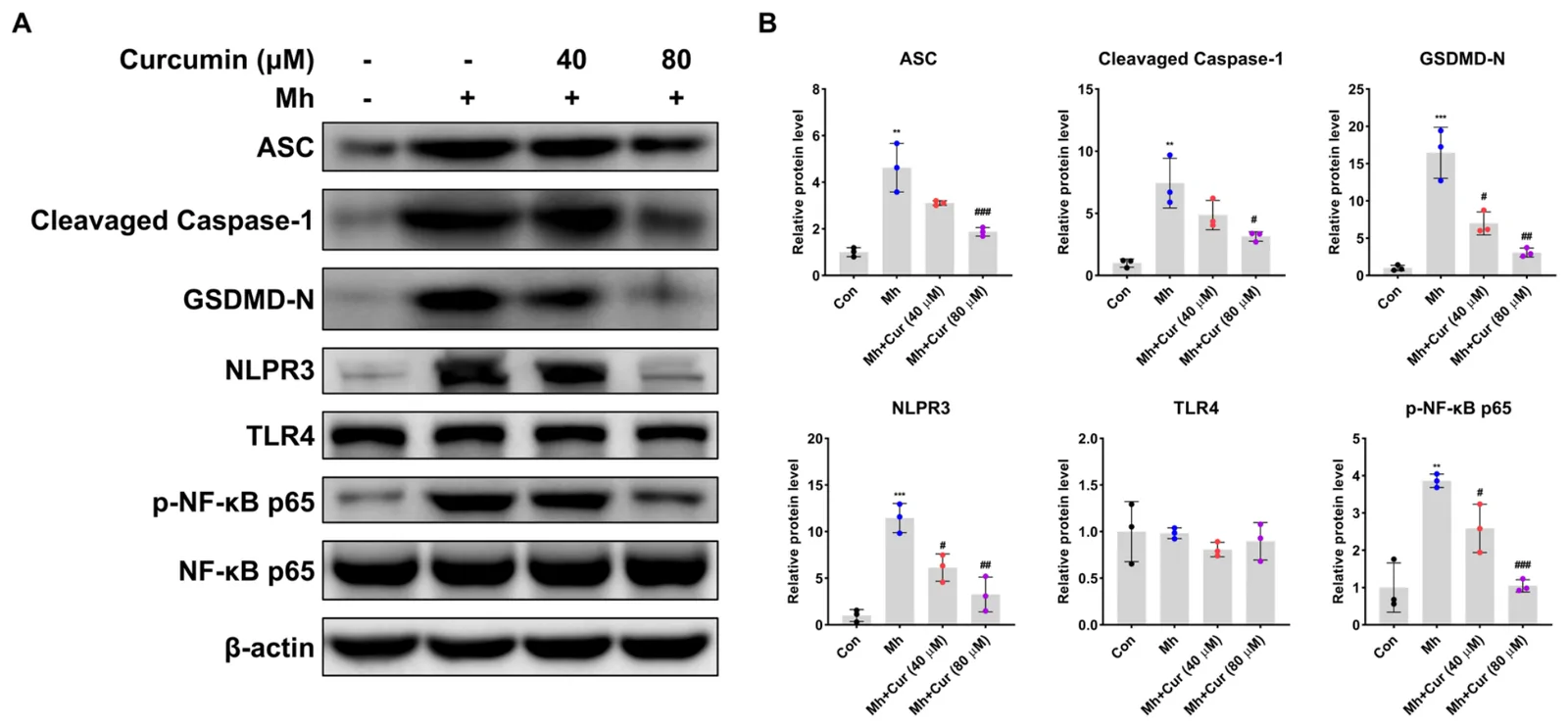
Curcumin pretreatment is associated with reduced inflammation-related protein changes in Mh-exposed BT cells. (**A**) Immunoblots for ASC, cleaved caspase-1, GSDMD-N, NLRP3, TLR4, phosphorylated NF-κB p65, total NF-κB p65, and β-actin. (**B**) Densitometric analysis. Three independently conducted cell-culture experiments were analyzed. Data are means ± SDs. ** *p* < 0.01 and *** *p* < 0.001 versus Con; # *p* < 0.05, ## *p* < 0.01, and ### *p* < 0.001 versus Mh.

In BT cells, viability declined as the Mh concentration increased, and 1 × 10^6^ CFU/mL was selected for the challenge condition (Figure S1A). Curcumin concentrations of 40 and 80 μM were selected from the viability screen (Figure S1B). Consistent with the animal data, curcumin pretreatment was associated with lower ASC, cleaved caspase-1, GSDMD-N, NLRP3, and phosphorylated NF-κB p65 levels after Mh exposure (Figure 5A,B). These results provide complementary bovine-cell evidence but remain association-based.

### 3.4. Curcumin Pretreatment Is Associated with Nrf2-Related Antioxidant Protein Changes

**Figure 6:**
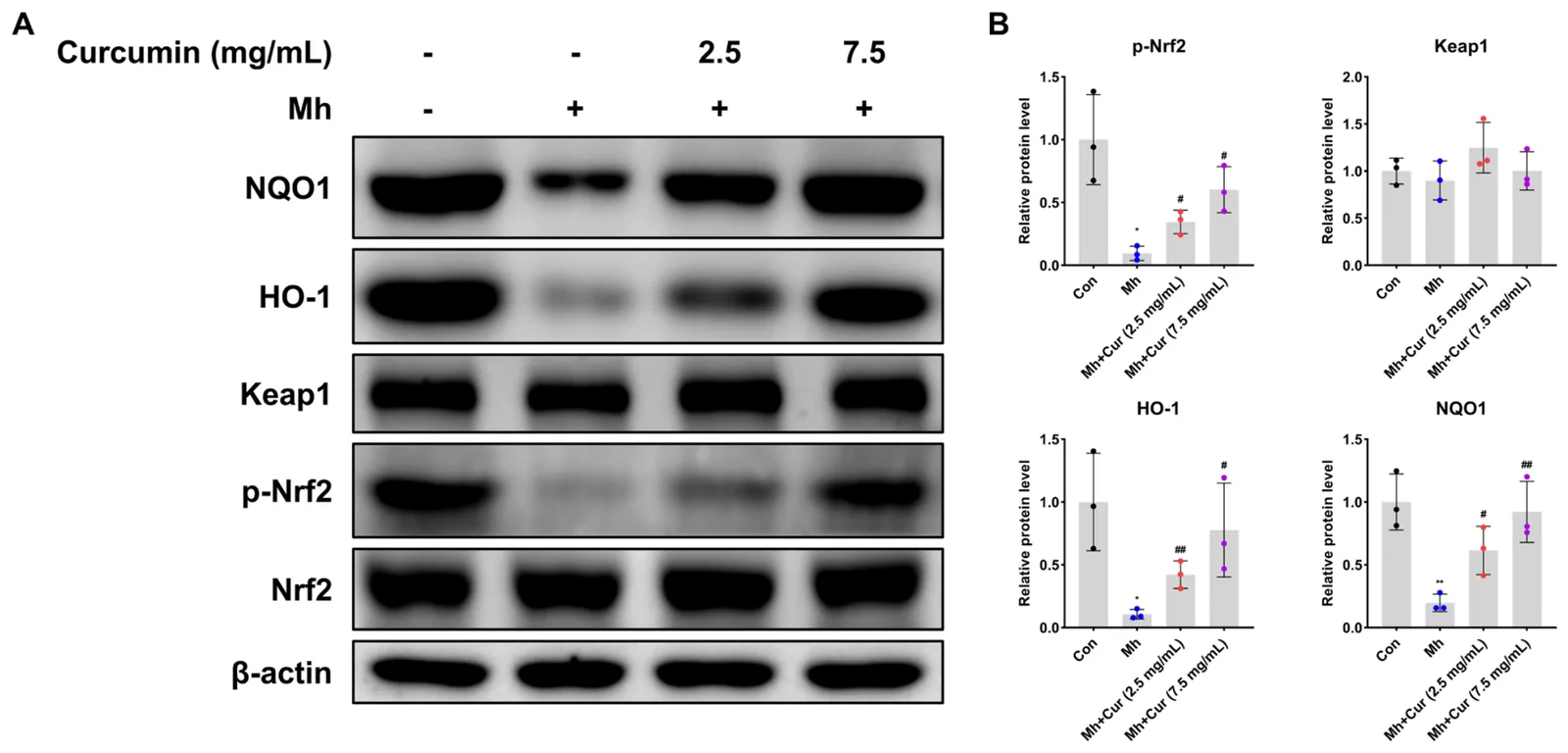
Curcumin pretreatment is associated with Nrf2-related antioxidant protein changes in lung tissue. (**A**) Immunoblots for NQO1, HO-1, Keap1, phosphorylated Nrf2, total Nrf2, and β-actin. (**B**) Densitometric analysis. NQO1, HO-1, and Keap1 were normalized to β-actin; phosphorylated Nrf2 was normalized to total Nrf2. n = 3 independent animal-derived biological samples/group. Data are means ± SDs. * *p* < 0.05 and ** *p* < 0.01 versus Con; # *p* < 0.05 and ## *p* < 0.01 versus Mh.

The Mh challenge was associated with lower NQO1, HO-1, and phosphorylated Nrf2 levels in lung tissue. Curcumin pretreatment was associated with higher levels of these Nrf2-related antioxidant proteins (Figure 6A,B). Because no Nrf2 perturbation or nuclear-translocation experiment was performed, these data support an association with Nrf2-related antioxidant responses rather than direct proof of Nrf2 activation.

### 3.5. Curcumin Pretreatment Is Associated with Reduced Apoptosis

**Figure 7:**
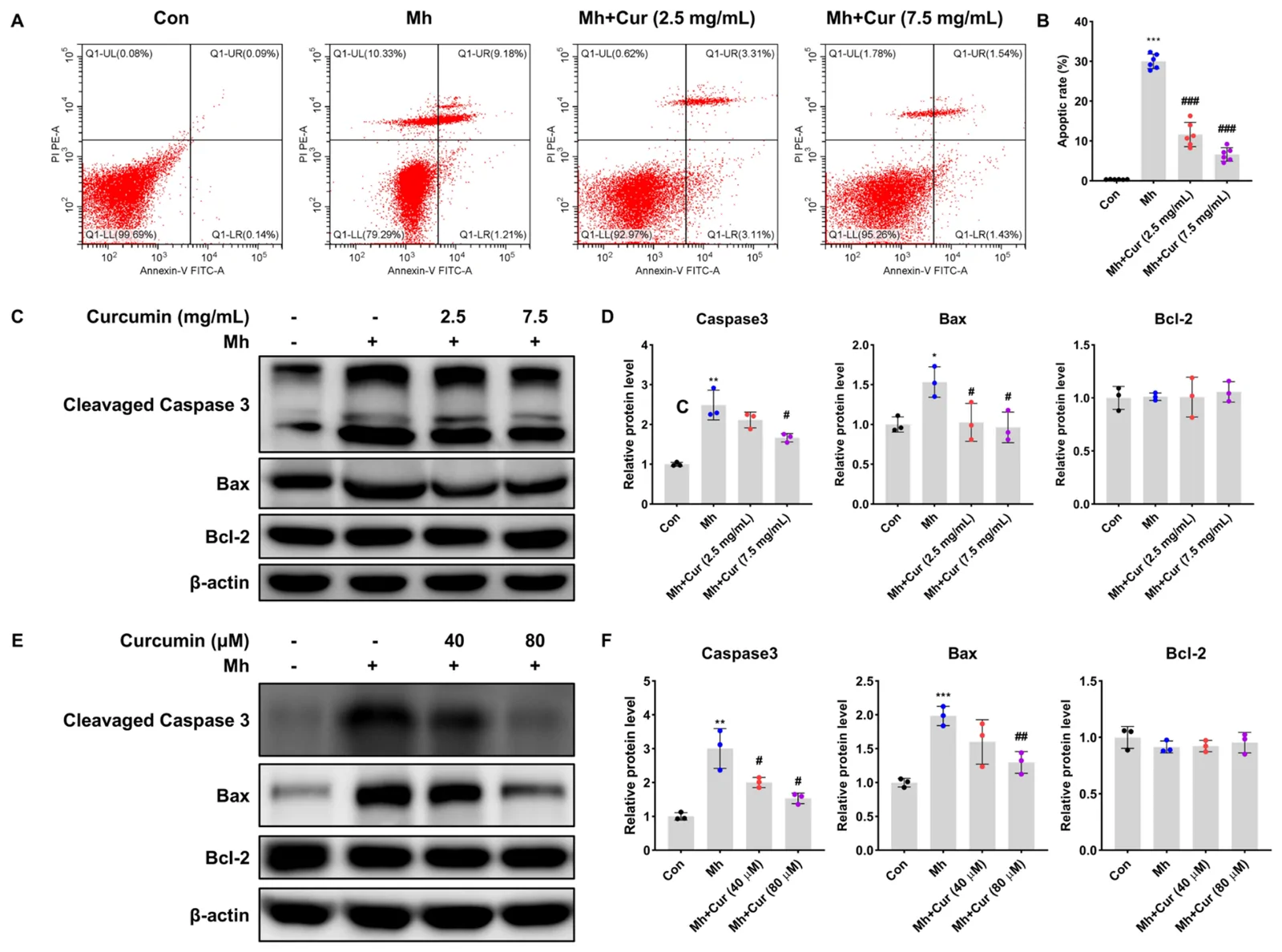
Curcumin pretreatment is associated with reduced apoptosis after Mh challenge. (**A**) Annexin V/PI flow-cytometry plots. (**B**) Apoptotic-cell percentage. (**C**,**E**) Immunoblots for cleaved caspase-3, Bax, Bcl-2, and β-actin in lung tissue and BT cells. (**D**,**F**) Densitometric analysis. For in vivo analyses, n = 3 independent animals/group; in vitro analyses were based on three independent cell-culture experiments. Data are means ± SDs. * *p* < 0.05, ** *p* < 0.01, and *** *p* < 0.001 versus Con; # *p* < 0.05, ## *p* < 0.01, and ### *p* < 0.001 versus Mh.

Annexin V/PI analysis showed a higher apoptotic-cell percentage after the Mh challenge and a lower percentage in the curcumin-pretreated groups (Figure 7A,B). Mh also increased Bax and cleaved caspase-3, whereas curcumin pretreatment was associated with lower levels of these pro-apoptotic proteins in lung tissue and BT cells (Figure 7C–F). Bcl-2 showed comparatively smaller changes.

### 3.6. Curcumin Pretreatment Is Associated with Preservation of Epithelial Barrier Proteins

**Figure 8:**
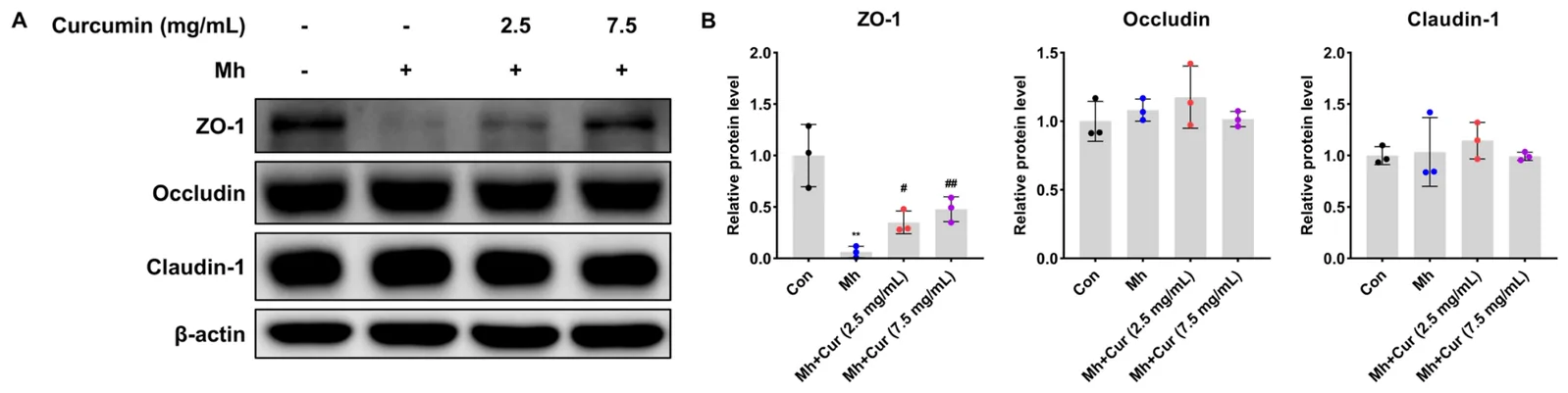
Curcumin pretreatment is associated with preservation of epithelial barrier proteins after Mh challenge. (**A**) Immunoblots for ZO-1, occludin, claudin-1, and β-actin. (**B**) Densitometric analysis normalized to β-actin. n = 3 independent animal-derived biological samples/group. Data are means ± SDs. ** *p* < 0.01 versus Con; # *p* < 0.05 and ## *p* < 0.01 versus Mh.

The Mh challenge reduced the abundance of the tight-junction protein ZO-1 in lung tissue. Curcumin pretreatment was associated with preservation of ZO-1 and more modest changes in occludin and claudin-1 (Figure 8A,B). These protein data, together with BALF protein measurements, are consistent with reduced epithelial-barrier injury but do not establish the specific molecular mechanism.

### 3.7. Curcumin Pretreatment Is Associated with Changes in Fecal Microbial Composition

**Figure 9:**
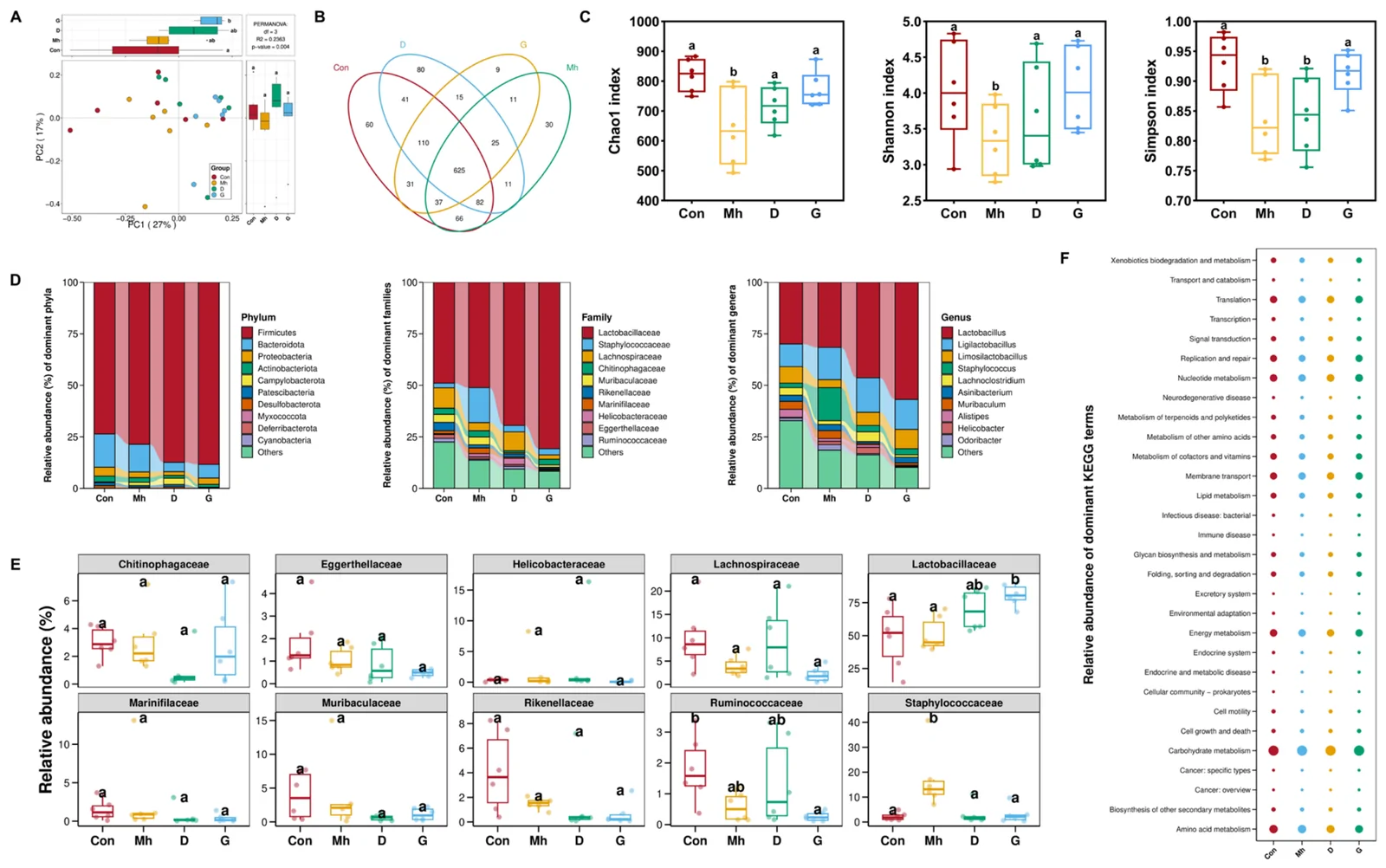
Curcumin pretreatment is associated with changes in fecal microbial composition. (**A**) Principal coordinate analysis with PERMANOVA. (**B**) Shared and unique OTUs among groups. (**C**) Chao, Shannon, and Simpson indices. (**D**) Relative composition at phylum, family, and genus levels. (**E**) Relative abundance of the 10 most abundant families. (**F**) PICRUSt2-predicted KEGG level-2 functional categories. n = 8 independent fecal samples/group, with one sample per mouse. PICRUSt2 results represent predicted functional potential and not direct metabolic measurements. Different letters indicate significant differences.

Fecal 16S rRNA sequencing showed separation among groups in principal coordinate analysis (PERMANOVA *p* = 0.004; Figure 9A) and group-associated differences in observed OTUs and alpha-diversity indices (Figure 9B,C). Curcumin pretreatment was associated with changes in bacterial composition at the phylum, family, and genus levels (Figure 9D,E and Figure S2), including higher Lactobacillaceae and lower Staphylococcaceae relative abundance than in the Mh group. PICRUSt2 analysis identified differences in predicted functional categories (Figure 9F); these are computational inferences and do not demonstrate actual microbial metabolic activity.

### 3.8. Curcumin Pretreatment Is Associated with Changes in Intestinal Short-Chain Fatty Acids

**Figure 10:**
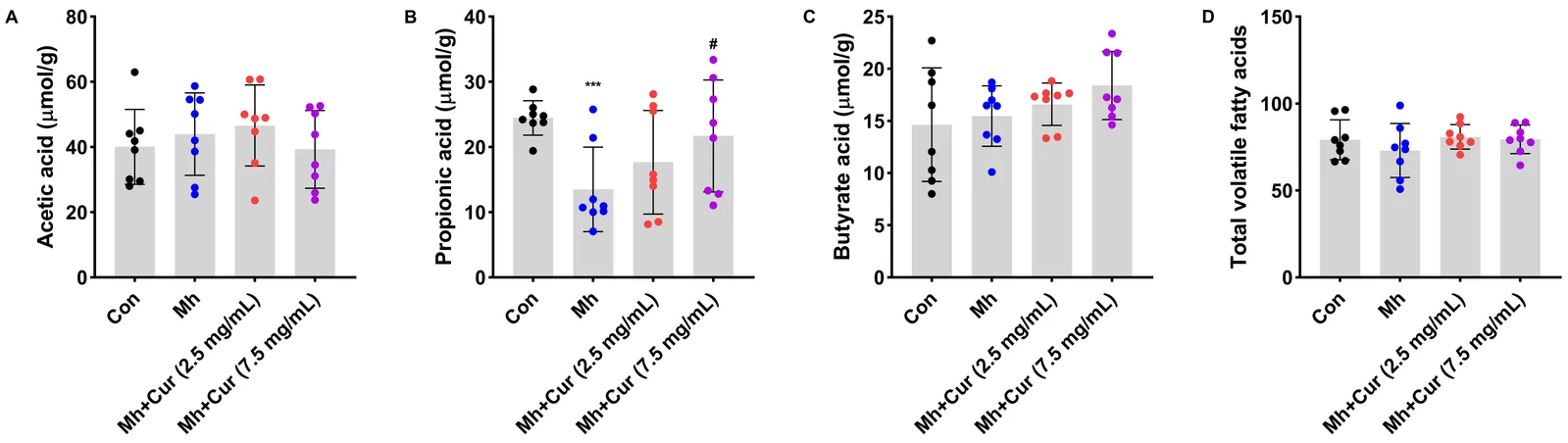
Curcumin pretreatment is associated with changes in intestinal short-chain fatty acids after Mh challenge. Intestinal acetic acid (**A**), propionic acid (**B**), butyric acid (**C**), and total volatile fatty acids (**D**) were quantified by GC-MS using authentic standards and 2-ethylbutyric acid as the internal standard and were normalized to tissue wet weight. n = 6 independent animals/group. Data are means ± SDs. *** *p* < 0.001 versus Con; # *p* < 0.05 versus Mh.

GC-MS analysis of intestinal samples showed that the Mh challenge was associated mainly with a reduction in propionic acid, whereas curcumin pretreatment was associated with partial recovery of propionic acid (Figure 10A–D). Acetic acid, butyric acid, and total volatile fatty acids showed smaller or less consistent group differences. These data establish an association between pretreatment and intestinal SCFA profiles but do not demonstrate that SCFAs mediated the pulmonary effects.

## 4. Discussion

This study examined seven days of curcumin pretreatment in a mouse model evaluated 12 h after an Mh challenge. The data show that pretreatment was associated with less early pulmonary edema and histological injury, lower systemic inflammatory markers, reduced inflammation- and apoptosis-related protein changes, preservation of selected epithelial junction proteins, and altered fecal microbiota and intestinal SCFA profiles. The findings should be interpreted as prophylactic host-protective associations rather than evidence of antibacterial activity or treatment of established pneumonia.

M. haemolytica is primarily a ruminant animals pathogen, and a mouse model cannot reproduce bovine lung anatomy, leukotoxin susceptibility, respiratory immunity, host–pathogen interactions, or gut microbiota. Nevertheless, the model provides a controlled system for studying early edema, inflammatory injury, apoptosis, and barrier disruption after an intranasal challenge, and the complementary BT-cell experiments increase respiratory-epithelial relevance. Direct extrapolation to cattle is not justified, and validation in calves or bovine respiratory infection models is required [23].

Curcumin pretreatment reduced the lung wet-to-dry ratio, BALF protein, histological injury, and serum pro-inflammatory cytokines. These findings are consistent with reports that curcumin can attenuate inflammatory and endothelial-barrier injury in other acute lung-injury models [24]. However, lung, BALF, blood, and tissue bacterial loads were not quantified. Consequently, the study cannot determine whether curcumin altered Mh colonization or clearance, and the observed protection may reflect improved host tolerance rather than antimicrobial activity.

The lower levels of phosphorylated NF-κB p65, ASC, cleaved caspase-1, GSDMD-N, and NLRP3 after curcumin pretreatment are compatible with reduced NF-κB/NLRP3-related activation [25,26,27]. Similarly, higher NQO1, HO-1, and phosphorylated Nrf2 levels are compatible with greater Nrf2-related antioxidant responses [28,29]. Because the study did not use pathway inhibitors, genetic manipulation, nuclear-translocation assays, or rescue experiments, these protein changes cannot establish direct pathway causality.

Curcumin pretreatment was also associated with lower Annexin V-positive cell percentages, Bax, and cleaved caspase-3, as well as preservation of ZO-1. These observations support reduced apoptosis and barrier injury in the early challenge phase [17,18,30,31,32,33,34,35,36]. Nevertheless, the molecular sequence linking inflammatory, antioxidant, apoptotic, and junctional changes was not directly tested.

The fecal microbiota and intestinal SCFA findings provide an exploratory view of systemic changes accompanying an early Mh challenge. Curcumin pretreatment was associated with higher microbial diversity, shifts in selected bacterial families, and partial recovery of propionic acid. Serum D-lactate also changed among groups. However, the study did not include a curcumin-only group, longitudinal baseline sampling, fecal microbiota transplantation, antibiotic depletion, germ-free animals, metagenomics, targeted microbial-function validation, or SCFA-blocking experiments. Therefore, the data do not show that microbiota or SCFAs caused the pulmonary protection, and PICRUSt2 results should be viewed only as predicted functional potential [8,9,37,38,39,40].

A possible advantage of curcumin is that one compound may simultaneously influence inflammatory, oxidative, apoptotic, and barrier-associated responses. This multi-target profile differs conceptually from interventions directed at a single inflammatory or oxidative target. However, the present study did not compare curcumin with approved antibiotics, non-steroidal anti-inflammatory drugs, corticosteroids, or other antioxidants, so no claims regarding superiority, additive benefits, safety, or dose equivalence can be made.

Curcumin also has poor aqueous solubility and limited oral bioavailability. The present study used a saline suspension and did not assess pharmacokinetics, tissue exposure, formulation stability, or long-term safety. These factors may substantially affect translation to cattle and should be addressed through formulation and exposure studies before practical application.

This study has several additional limitations. It lacked a curcumin-only group, a post-infection treatment group, later disease time points, local BALF or lung-tissue cytokine measurements, bacterial-burden measurements, pathway-perturbation experiments, and causal microbiota experiments. The 12 h endpoint represents an early acute response rather than established pneumonia. Full-length immunoblot images and detailed sequencing-read-depth, rarefaction, and coverage summaries were not available in the submitted Supporting Information; accordingly, the Western blot and microbiota conclusions have been narrowed. These limitations should be considered when interpreting the findings.

## 5. Conclusions

In this prophylactic mouse model study, a seven-day curcumin pretreatment was associated with attenuation of early Mh-induced pulmonary injury and with coordinated changes in systemic inflammatory markers, NF-κB/NLRP3-related proteins, Nrf2-associated antioxidant proteins, apoptosis, epithelial junction proteins, fecal microbial composition, and intestinal SCFAs. This study does not establish antibacterial activity, post-infection therapeutic efficacy, direct pathway causality, or microbiota-mediated protection. Curcumin should therefore be considered a candidate multi-target preventive adjunct for further evaluation rather than a proven treatment for ruminant animals Mannheimia pneumonia.

## Data Availability

The original contributions presented in this study are included in the article/Supplementary Material. Further inquiries can be directed to the corresponding authors.

## Supplementary Materials

The following supporting information can be downloaded at https://www.mdpi.com/article/10.3390/microorganisms14081764/s1.

## Abbreviations

Mh	Mannheimia haemolytica
BRDC	Bovine respiratory disease
Cur	Curcumin
BALF	Bronchoalveolar lavage fluid
SCFA	Short-chain fatty acid
BT	Bovine turbinate
