# Supplementary material for: Curcumin Pretreatment Alleviates Early Mannheimia Haemolytica-Induced Pulmonary Injury in Mice and Is Associated with Changes in Gut Microbial Composition
Source: Microorganisms. 2026 Aug 11;14(8):1764. doi: 10.3390/microorganisms14081764 (PMC13515592; doi:10.3390/microorganisms14081764)
Supplement: Supplementary file 1 [file microorganisms-14-01764-s001.zip › microorganisms-4392084-supplementary.pdf]

Curcumin Pretreatment Alleviates Early Mannheimia Haemolytica-Induced Pulmonary Injury in Mice and Is Associated with Changes in Gut Microbial Composition

Fangfang Zhao [1,†], Yixin Huang [1,†], Shifeng Wu [1], Yong Fu [2], Xueyong Zhang [2], Zhi Li [2], Xin An [2], Kun Zhang [1], Liuhong Shen [1], Suizhong Cao [1], Huanrong Zhang [3], Xiuying Shen [2,*] and Shumin Yu [1,*]

[1] College of Veterinary Medicine, Sichuan Agricultural University, Chengdu 610041, China

[2] Qinghai Academy of Animal Science and Veterinary, Qinghai University, Xining 810016, China

[3] College of Animal and Veterinary Sciences, Southwest Minzu University, Chengdu 610041, China

* Correspondence: 1998990030@qhu.edu.cn (X.S.); yayushumin@sicau.edu.cn (S.Y.)

[†] These authors contributed equally to this work.

Supporting Information

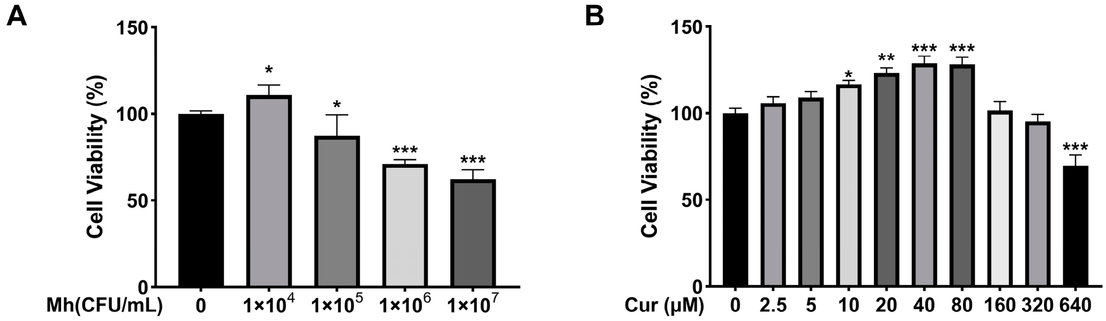

Figure S1. Selection of Mh and curcumin concentrations for BT-cell experiments. (A) BT-cell viability after exposure to the indicated Mh concentrations. (B) BT-cell viability after exposure to the indicated curcumin concentrations. Data were normalized to untreated controls.

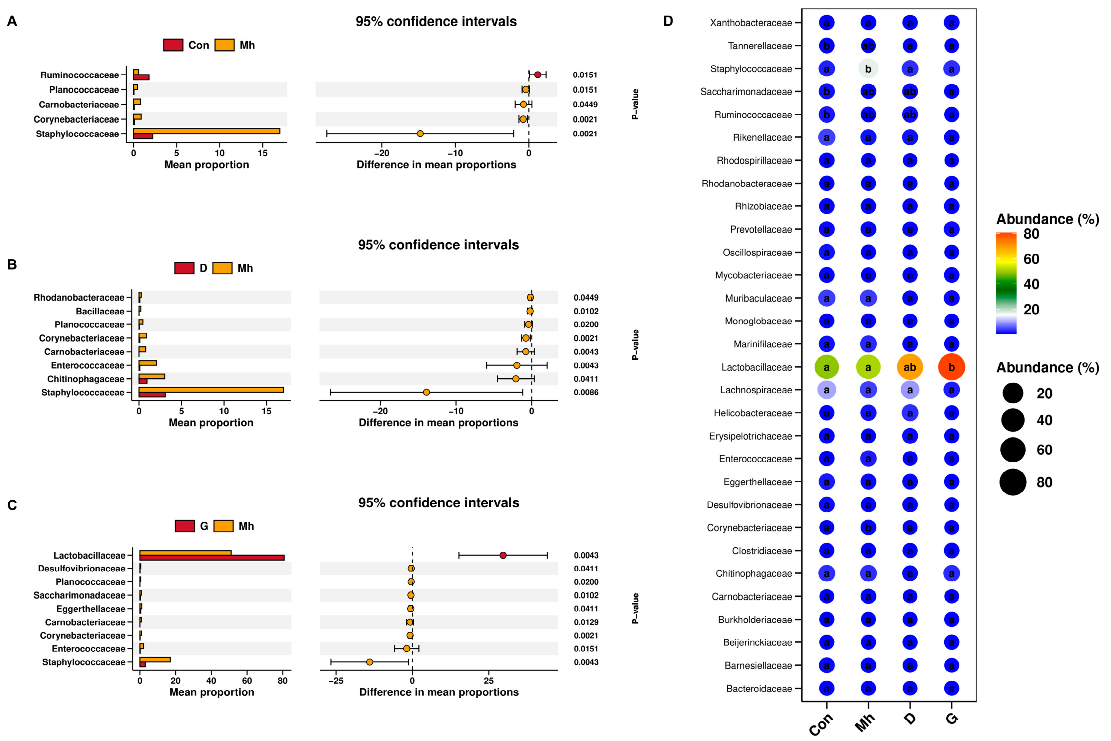

Figure S2. Curcumin pretreatment is associated with changes in fecal bacterial abundance at the family level. (A) Con versus Mh. (B) Mh versus Cur-D. (C) Mh versus Cur-G. (D) Relative abundance of selected bacterial families among the four groups shown as a bubble plot. These analyses describe compositional associations and do not establish microbiota-mediated causality.

Note S1. Scope of the microbiota analysis. The 16S rRNA analysis used individual fecal samples from n = 8 mice/group. Each sample represented one biological replicate. PICRUSt2 results represent inferred functional potential based on 16S composition and do not directly measure microbial transcription or metabolism. No fecal microbiota transplantation, antibiotic-depletion, germ-free, or SCFA-blocking experiments were performed.

Materials and Methods

MTT assay

BT cells were seeded in 96-well plates at 5×10^4 cells/well. After 24 h, cells were exposed to the indicated concentrations of curcumin or Mh for 48 h. MTT solution (10 μL; 5 mg/mL) was then added for 4 h. Formazan crystals were dissolved in 100 μL DMSO for 10 min, and absorbance was measured at 490 nm. Cell viability was expressed relative to untreated controls. Independent experiments used separately seeded cell cultures; technical wells within each experiment were averaged before statistical analysis.
